# Donor-Specific Antibodies Targeting a Repeated Eplet Mismatch and Outcome After Kidney Retransplantation

**DOI:** 10.3389/ti.2024.13639

**Published:** 2024-11-29

**Authors:** Caroline Arches, Cédric Usureau, Dany Anglicheau, Alexandre Hertig, Arwa Jalal-Eddine, Mohamad Zaidan, Jean-Luc Taupin, Renaud Snanoudj

**Affiliations:** ^1^ Faculté de Médecine, Sorbonne Universités, Paris, France; ^2^ Department of Nephrology-Dialysis-Transplantation, Bicêtre Hospital, Assistance Publique des Hôpitaux de Paris, Le Kremlin-Bicêtre, France; ^3^ Immunology and Histocompatibility Laboratory, Saint-Louis Hospital, Assistance Publique des Hôpitaux de Paris, Paris, France; ^4^ Department of Nephrology and Transplantation, Necker Hospital, Assistance Publique des Hôpitaux de Paris, Paris, France; ^5^ Department of Nephrology, Foch Hospital, Suresnes, France; ^6^ Institut National de la Santé et de la Recherche Médicale U1184 Centre de Recherche en Immunologie des Infections Virales et des Maladies Auto-Immunes, Le Kremlin-Bicêtre, France

**Keywords:** kidney transplantation, antibody-mediated rejection, donor-specific antibody, eplets, graft outcomes

## Abstract

Kidney retransplantations are associated with an increased risk of rejection and reduced graft survival compared to first transplantations, notably due to HLA sensitization. The impact of repeated eplet mismatches on retransplantation outcome has not been investigated. We retrospectively assessed the risk of antibody-mediated rejection (ABMR) and graft loss associated with preformed DSA targeting Repeated Eplet MisMatches (DREMM) in sensitized patients undergoing kidney retransplantation. We included 45 retransplanted patients with preformed DSA against the second donor. We determined HLA incompatibilities at the eplet levels, and the eplet target of the DSA using HLAMatchmaker^®^. Repeated mismatches were more frequent at the eplet (87%) than at the antigenic level (22%), but were not associated with the risk of ABMR. The eplet specificity of the DSA revealed that 60% of patients (n = 27) had DREMM. The presence of DREMM was associated with a higher frequency of ABMR (70% versus 28%, *P* = 0.005) and with a lower death-censored graft survival (log-rank test, *P* = 0.01). However, in multivariate Cox model, we could not show that DREMM were associated with the risk of ABMR. In conclusion, this study suggests that identifying DREMM may be an interesting clinical tool, however further larger studies are necessary to precise their exact predictive value.

## Graphical Abstract

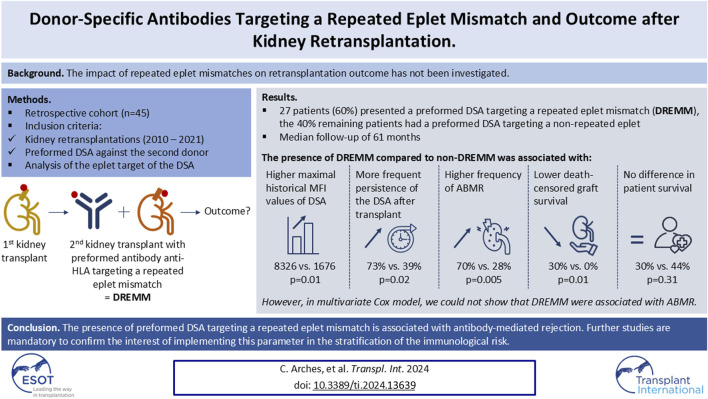

## Introduction

Long-term renal allograft survival rates remain unsatisfactory, only reaching 50%–60% survival at 10 years [1]. Therefore, a growing number of patients require a repeat transplantation, currently representing 22% of the patients of the French waiting list [2]. These patients are more susceptible to be immunized due to previous alloantigens exposure, particularly HLA, during the first transplant [2]. This HLA sensitization represents a major risk factor for both rejection and graft loss, mainly due to the development of a humoral memory and anti-HLA antibodies [3–5]. As a result, these patients experience longer waiting times for a compatible kidney transplant and poorer outcomes when retransplanted [6, 7].

So, in case of retransplantation it is critical to assess the risk of rejection against the second donor, inherited from the sensitization towards the first donor. It is known that for a first transplant, a high number of HLA antigenic, particularly class II, mismatches raises the risk of developing *de novo* anti-HLA donor specific antibodies (DSA) [8], rejection [9] and graft failure [10, 11]. Regarding retransplantation, several retrospective studies have investigated the effect of antigenic repeated HLA mismatches (RMM), defined as a mismatch presented by both the first and the second donor. However, the results are controversial regarding their impact on the risk of rejection and on the graft survival [12–20]. A recent study found that an antigenic RMM increased the risk of antibody-mediated rejection (ABMR) and graft failure only in case of a preformed DSA targeting this RMM [20].

Although mismatches are commonly determined at the antigenic level, allelic high-resolution HLA typing is now easily accessible and provides precise data on the HLA molecular structure, allowing identification of the different B-cell eplets presented by donor HLA-incompatible molecules. Eplets represent the functional part of the epitopes, which are the zone on the antigens in contact with the antibody paratopes, and which correspond to one or a few amino acid residues in a location on the antigen surface accessible to antibodies. Given that anti-HLA antibodies recognize eplets rather than the whole HLA antigens, several studies have assessed the impact of eplet mismatches in kidney transplant recipients. A high number of eplet mismatches is associated with an increased risk of DSA *de novo* synthesis [21–23], rejection [22–24], and graft loss [22, 23, 25, 26], with a better predictive value than antigenic mismatches.

There are limited data concerning the impact of eplet mismatches in kidney retransplantation or in sensitized patients. Two case reports have highlighted the role of shared eplets between the current donor and a previous immunizer in triggering ABMR [27, 28]. Additionally, a recent retrospective study showed that in a population of kidney transplant recipients with preformed DSA, some clinically relevant donor eplet-specific antibodies decreased graft survival [29]. Nevertheless, studies regarding B-cell eplet RMM between first and second donor after retransplantation are lacking.

The aim of this study is to assess the risk associated with preformed DSA directed against repeated eplet mismatches in sensitized patients undergoing a second kidney transplantation. In other words, the goal is to determine whether humoral sensitization driven by eplets of the first donor affects the outcome of the second transplant if the DSA against the second donor targets these eplets.

## Materials and Methods

### Study Population

This retrospective study included all patients who underwent a second kidney transplantation between 2010 and 2021 with preformed DSA against the second donor, at two French transplantation centers: Necker-Enfants Malades and Foch Hospitals. We considered DSA against HLA-A, -B, -C, -DRB1, -DRB3/4/5, -DQA1 and -DQB1 loci, but not DPB1 or DPA1, with a Mean Fluorescence Intensity (MFI) ≥ 500. We excluded the patients whose follow-up was less than 6 months, those without complete HLA typing of the first donor and the patients whose analysis of DSA eplet specificity could not identify any target. The study was done in accordance with the Declaration of Helsinki. The clinical and research activities being reported are consistent with the Principles of the Declaration of Istanbul as outlined in the “Declaration of Istanbul on Organ Trafficking and Transplant Tourism.” All patients provided written informed consent at the time of transplantation for anonymous collection of data for clinical research purposes.

### Data Collection

Clinical data were retrospectively retrieved from patients’ medical records, the DIVAT database,^1^ and from the national database of kidney transplant recipients and donors (Cristal).^2^ The results of all graft biopsies, screening and for-cause biopsies, have been collected for all patients. Each biopsy was assessed by an experienced nephropathologist, according to the Banff classification available at the time of biopsy. The conclusions were then reanalyzed according to the 2022 Banff classification for the diagnosis of ABMR [30]. All patients were followed-up from the day of the transplantation until the occurrence of graft loss, death, or the date of the final data extraction (29 December 2023).

### HLA Typing

The method of HLA typing depended on the transplantation period. For recipients before 2017 and all deceased donors, HLA typing was performed using polymerase chain reaction sequence-specific primers (Olerup SSP) and the results were provided with two-digits for HLA-A, -B, -DRB1, and -DQB1. For recipients since 2017 and living donors, HLA typing was done with sequence-specific oligonucleotide technology (PCR-SSO One Lambda) or next-generation sequencing for the same loci, and HLA-Cw and -DQA1. In the case where the typing provided a list of ambiguities, the most frequent allele was retained based on the most frequent haplotype. To complete loci without specific typing or for some ancient donors with no DNA remaining, we imputed the most likely high-resolution allelic typing (four-digit) from the two-digit resolution typing using the HaploSFHI tool. This algorithm was developed from data from a majority of the France histocompatibility laboratories and validated on European cohorts [31].

### Detection of DSA

Patients on the waiting list were systematically screened for anti-HLA antibodies before transplantation every 3 months. All sera were analyzed in the same laboratory (Immunology Laboratory of Saint-Louis Hospital, Paris) using the Luminex Labscreen screening assay, and if positive completed with the Single Antigen assay (both from One Lambda ThermoFisher Scientific, West Hills, CA). Loci tested were HLA-A, -B, -Cw, -DRB1, -DRB3/4/5, -DQA1, and -DQB1.

Preformed DSA were defined with an MFI above 500 after subtracting the background noise and the autoreactivity. The immunodominant DSA was defined as the DSA with the highest MFI value over all sera collected during the pretransplant period. *De novo* DSA were detected during the post-transplant course but not before transplantation.

### Determination of HLA Compatibility, Repeated Mismatches and Antibody-Targeted Eplets

HLA compatibility between the recipient and the second donor was assessed by the number of mismatches at the antigenic (assimilated to serological group or first-field DNA typing), allelic (assimilated to second-field DNA typing), and eplet level for HLA-A, -B, -Cw and HLA-DRB1, -DRB3/4/5, DQB1 and DQA1 (only at the allelic and eplet level). For the latter, we determined the mismatched eplets expressed by the first and the second donor but not by the recipient, from the eplet database of the HLAMatchmaker Antibody Analysis software (version 3.1).^3^ Mismatched eplets shared by the two donors were identified as eplet repeated mismatches. We focused on antibody-verified eplets, i.e., eplets verified by analyzing reactivity patterns of either polyclonal sera or monoclonal antibodies and updated in the HLA Eplet Registry.^4^

We used the HLAMatchmaker Antibody Analysis software to determine the targeted eplets by preformed DSA. We entered donor and recipient allelic typing into the software, the MFI values of all Luminex beads from the pre-transplant sera. The cut-off for MFI positivity was set at a minimum of 500 and sometimes higher, based on the average value of self-antigen beads.

Eplets expressed by the HLA molecules of the recipient and of the negative beads were excluded as potential candidates for targeted eplets. We determined which eplets expressed by the donor antigens targeted by DSA could explain the antibody reactivity pattern. When these eplets were repeated between the first and the second donor, the DSA was referred as “preformed DSA targeting a Repeated antibody-verified Eplet MisMatch (DREMM).”

### Statistical Analysis

Patient data were summarized using frequencies and percentages for categorical variables, and medians with interquartile ranges or means with standard deviations for continuous variables. We used the Wilcoxon rank-sum test to compare numeric data, and chi-square or Fisher’s exact tests for categorical data. The cumulative incidence of ABMR and non-death-censored graft loss considered as competitive risks were determined with the Aalen-Johansen estimator and compared in patients with and without DREMM (Package Survival).

Graft and patient survival analyses were performed using Kaplan‒Meier estimates and were compared between patients with or without DREMM using the log-rank test. Univariate and multivariate Cox proportional hazards models were applied using hazard ratios to identify immunological factors associated with the risk of ABMR and graft loss from the day of transplant. Multicollinearity between covariates was tested when necessary, with the package “Car”. All statistical analyses were performed using R software version 4.3.3 (R Foundation for Statistical Computing, Vienna, Austria). A two-sided *p*-value of <0.05 was considered statistically significant.

## Results

### Study Population

We screened 69 recipients who underwent a second kidney transplantation between 2010 and 2021 with preformed DSA and after applying exclusion criteria, we included 45 sensitized patients with a median follow-up of 5.1 years (Supplementary Figure S1). The first transplantation had been performed between 1982 and 2015, and patients received their second transplant after a median delay of 12 years. All recipients had preformed DSA explaining the use of thymoglobulin as induction therapy in 89% of patients. The patients were primarily on a triple maintenance immunosuppressive regimen including steroids, calcineurin inhibitor – mainly tacrolimus – and mycophenolic acid. Only one patient received a steroid-free regimen. Depending on local habits and characteristics of DSA, the immunosuppressive induction treatment for the second transplant also included, except for one recipient, at least one of the following: intravenous immunoglobulins, plasma exchanges, rituximab or eculizumab (Table 1).

**TABLE 1:** Clinical characteristics of the study population.

	Total n = 45
Recipients Age, years, median (IQR) Sex: female, n (%) Cause of ESRD, n (%) Diabetes Hypertension Polycystic disease Glomerular disease Interstitial nephritis Other Unknown	47 (35–56) 17 (38) 3 (7) 4 (9) 2 (4) 12 (27) 9 (20) 5 (11) 10 (22)
Immunosuppressive therapies for the 2nd transplantation Induction, n (%) Thymoglobulin IL-2 receptor blocker Initial maintenance therapy, n (%) Steroids + tacrolimus + MPA Steroids + cyclosporin + MPA Tacrolimus + MPA Associated treatments Intravenous immunoglobulin Plasma exchange Rituximab Eculizumab	40 (89) 5 (11) 42 (93) 2 (4) 1 (2) 40 (89) 30 (67) 16 (36) 3 (7)
Donors Age, years, median (IQR) Sex: female, n (%) Deceased donor, n (%)	47 (41–59) 23 (51) 39 (87)

IQR, interquartile range; ESRD, end-stage renal disease; DSA, donor specific antibody; IL, interleukin; MPA, mycophenolic acid.

### HLA Compatibility

The mean numbers of mismatches at the antigenic, allelic, and eplet (antibody-verified) level were 5.1 ± 2.3 (min-max: 1–10), 7.3 ± 3.2 [1–10] and 14.2 ± 7.2 [4–30], respectively (Supplementary Table S1). Whatever the level, the number of mismatches was lower for class II compared to class I. In total, 18 (40%) patients had no antibody-verified class II eplet mismatches, compared to only 1 (2%) patient for class I.

Subsequently, we focused on the identification of repeated mismatches (RMM) at these three levels between the first and the second donor (Table 2). RMM were more frequently evidenced at the eplet level since 10 (22%) recipients were exposed to an antigenic RMM, 13 (29%) to an allelic RMM and 39 (87%) to an eplet RMM during the second transplant. In addition, the eplet level allowed a finer stratification of mismatches with a wider range of RMM observed per patient: [0–13] at the eplet level versus [0–2] at the antigenic level and [0–4] at the allelic level. Class I antigenic and allelic RMM only concerned the HLA-Cw locus whereas eplet RMM involved the three class I loci.

**TABLE 2:** Repeated HLA mismatches at the antigenic, allelic and eplet level.

HLA repeated mismatches (RMM)	Missing and imputed typing R/D1/D2	Total n = 45
**Antigenic RMM**, number of patients (%) Number of RMM per patient* 0 1 2 Locus, number of patients A B C DR DQ*	0 0 17/36/3 0 0/1*/0	10 (22) 31 4 4 0 0 5 6 2
**Allelic RMM**, number of patients (%) Number of RMM per patient* 0 1 2 ≥3 Locus, number of patients A B C DR DQ*	8/43/40 7/41/31 17/41/30 8/41/37 7/35*/34	13 (29) 29 5 2 3 0 0 3 9 4
**Eplet RMM (AbV)**, number of patients (%) Number of RMM per patient* 0 1–3 4–6 ≥7 Locus, number of patients A B C DR DQ*	8/43/40 7/41/31 17/41/30 8/41/37 7/35*/34	39 (87) 5 18 9 7 14 19 25 15 13

*for 6 first donors, DQB1 typing was missing and could not be imputed; so the number of imputed DQB1 typing applies here to 39 donors.

R, recipient; D1, first donor; D2, second donor; RMM, repeated mismatch; AbV, antibody-verified.

**FIGURE 1:**
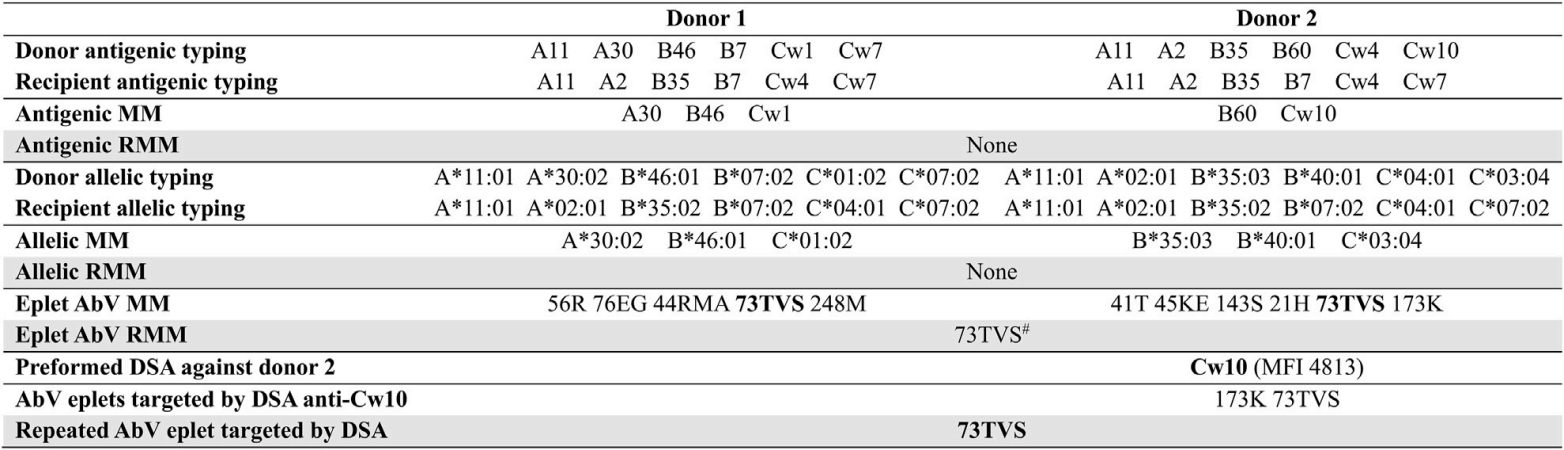
Example of the methodology used for the analysis of repeated mismatches and reactivity. There was no class II DSA. MM, mismatches; RMM, repeated mismatch; AbV, antibody-verified. ^#^shared eplet between B46, Cw1 and Cw10.

Figure 1 illustrates an example of the three levels of class I compatibility for one recipient and his two consecutive donors, and the analysis of the eplet reactivity of the preformed DSA against the second donor. At the antigenic and allelic levels, there was no RMM. Antibody-verified eplet load was higher towards the second donor (6 versus 5) despite fewer antigenic mismatches, highlighting the inconstant correlation between antigenic and eplet levels. Although there was no antigenic or allelic RMM, an antibody-verified eplet RMM (73TVS) was present due to the shared expression of this eplet by HLA B46 and Cw1 (1st donor), and Cw10 (2nd donor) molecules. The recipient had a preformed anti-Cw10 DSA. Analyzing the Luminex Single Antigen bead profile with HLAMatchmaker showed that this anti-Cw10 DSA was directed against the 73TVS antibody-verified eplet RMM.

### Characteristics of DSA Targeting a Repeated Eplet Mismatch

The study population was divided into two groups based on the presence or absence of preformed DSA targeting a Repeated antibody-verified Eplet MisMatch (DREMM). Twenty-seven (60%) recipients presented a DREMM whereas 18 (40%) did not. No difference was observed between the two groups regarding the class or locus of the highest preformed DSA, which was mainly class I (69%) (Table 3). The interval between the two transplants and the frequency of transplantectomy were similar in the two groups. The DREMM was isolated in 15 of the 27 patients (56%) or associated to one or more other DREMM in the 12 remaining patients (44%). The DREMM was the immunodominant DSA in 22 of the 27 cases. In the peak serum, i.e., the serum with the DSA of highest MFI between the two transplants, the MFI of the highest DSA and the sum of DSA MFI were significantly higher for DREMM compared to non-DREMM (median: 8,326 versus 1,676, *P* = 0.01 and 14,249 versus 2,482, *P* < 0.001, respectively).

**TABLE 3:** Characteristics of preformed DSA against the second donor.

	Total n = 45	No DREMM n = 18	DREMM n = 27	*P*-value
Locus of the preformed DSA with the maximal value, n (%) Class I A B Cw Class II DR DQB DQA	31 (69) 8 (26) 8 (26) 15 (48) 14 (31) 8 (57) 4 (29) 2 (14)	14 (78) 3 (21) 3 (21) 8 (57) 4 (22) 3 (75) 1 (25) 0 (0)	17 (63) 5 (29) 5 (29) 7 (41) 10 (37) 5 (50) 3 (30) 2 (20)	0.29
MFI_max_ value of the preformed DSA, median (IQR)	4,813 (1,566–11,667)	1,676 (1,146–6,407)	8,326 (2,880–12,928)	0.01
Sum of DSA MFI, median (IQR)	7,328 (2,667–16,352)	2,482 (1,292–6,787)	14,249 (7,163–24,752)	<0.001
Persistence of the preformed DSA with the maximal value, n (%) At 3 months post-transplant^a^ At 12 months post-transplant^b^	26 (59) 18 (43)	7 (39) 5 (28)	19 (73) 13 (54)	0.02 0.09
Time between 1st and 2nd transplant, years, median (IQR)	12 (9–16)	11 (9–14)	12 (10–17)	0.57
History of transplantectomy, n (%)	21 (46.7%)	6 (33.3)	15 (55.6)	0.14

DSA, donor-specific antibody; DREMM, donor-specific antibody targeting a repeated antibody-verified eplet mismatch; MFI, mean fluorescence intensity; Sum of MFI, sum of the MFI of each DSA the day of the maximal MFI of the preformed DSA.

^a^ missing value for one patient in the DREMM group.

^b^ missing values for three patients in the DREMM group.

**FIGURE 2:**
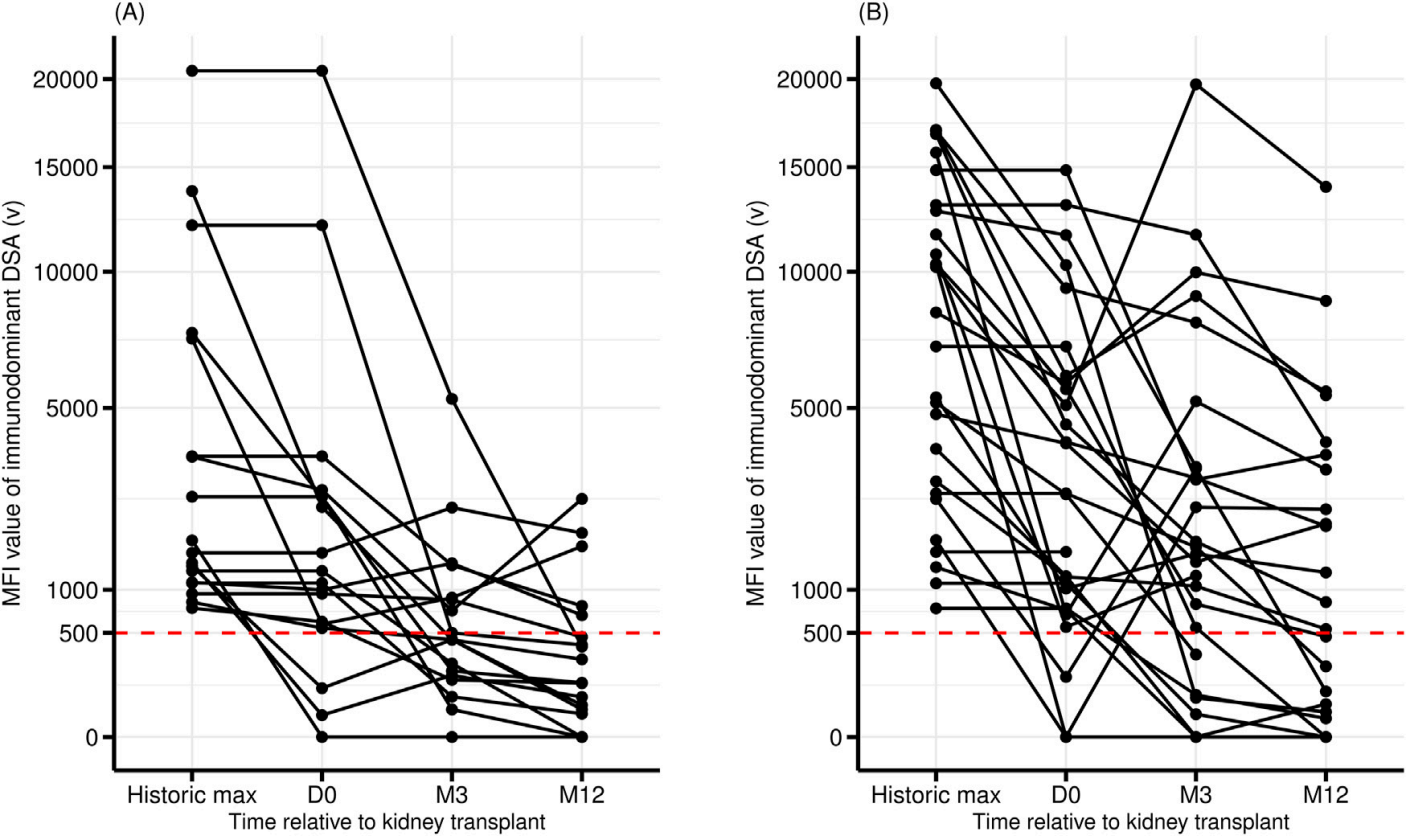
Pre- and post-transplant evolution of the MFI of the DSA with the highest MFI. DSA targeting a non-repeated eplet mismatch (no DREMM, n = 18) **(A)**, or a repeated antibody-verified eplet mismatch (DREMM, n = 27) **(B)**. MFI are represented as square roots.

The post-transplant evolution of the DSA also differed significantly depending on whether it targeted an eplet RMM or not (Table 3 and Figure 2). A significantly higher proportion of DREMM persisted at three but not at 12 months compared to non-DREMM [73% versus 39% (at month −3), *P* = 0.02, 54% versus 28% (at month −12), *P* = 0.09]. More DREMM experienced a significant increase (greater than 500 units) of their MFI between day-0 and month −3 compared to no-DREMM but this difference was not significant (31% versus 5%, *P* = 0.06) (Figure 2).

*De novo* DSA appeared in 3 (7%) patients, all in the DREMM group, within a median (IQR) time of 19 months (17.8–43.1). Only one of them experienced an ABMR episode after the occurrence of this *de novo* DSA.

### Association Between DSA Against a Repeated Eplet Mismatch, ABMR and the Risk of Graft Loss

**FIGURE 3:**
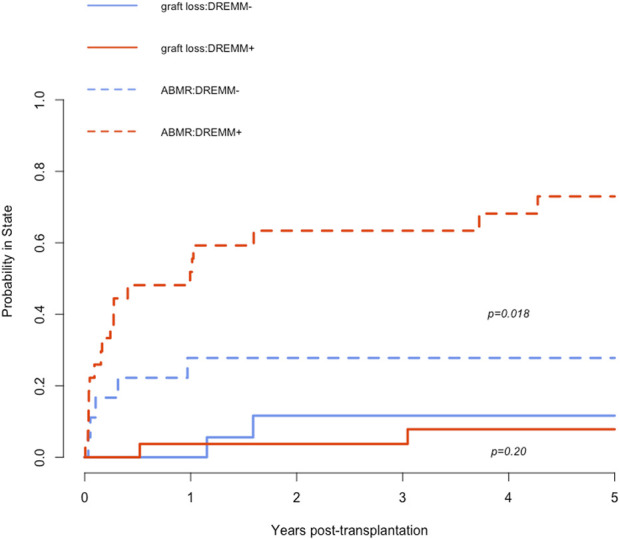
Cumulative incidence of ABMR and of overall graft loss as competitive risks in patients with and without DREMM. The dashed red curve corresponds to the incidence of ABMR in patients with DREMM, the dashed blue curve to patients without DREMM. The solid red curve corresponds to the incidence of overall graft loss before occurrence of any ABMR in patients with DREMM, the solid blue curve to patients without DREMM. DREMM, donor-specific antibody targeting a repeated antibody-verified eplet mismatch; ABMR, antibody-mediated rejection.

Among the 45 patients with preformed DSA, 24 (53%) patients developed an active ABMR during their follow-up. The presence of DSA directed against an antigenic RMM was not associated with an increased risk of ABMR (57% versus 43%; *P* = 1). However, patients with DREMM had significantly higher rates of ABMR during follow-up compared to patients without DREMM [19/27 (70%) versus 5/18 (28%); *P* = 0.005] (Table 4). The increase in the risk of ABMR associated with DREMM was statistically significant when these DSA targeted a class I eplet RMM but not a class II eplet RMM. We compared the cumulative incidence of ABMR and of overall graft loss as competitive risks in patients with and without DREMM (Figure 3). These graft losses, occurring before any ABMR episode, mainly consisted in the death of patients (n = 7) and in death-censored graft loss in one patient. Patients with DREMM had a higher cumulative incidence of ABMR (HR 3.37 [1.25–9.06], *P* = 0.018), but not of overall graft loss (HR 2.08 [0.85–5.1], *P* = 0.2).

**TABLE 4:** Outcome of patients with or without DREMM.

	Total n = 45	No DREMM n = 18	DREMM n = 27	*P*-value
Active ABMR, n (%) Subclinical/clinical (%/%) Time post-transplant, months, median (IQR) Associated TCMR, n (%) Chronic ABMR on subsequent biopsies, n (%)	24 (53) 29/71 2.4 (0.5–11.7) 2 (8) 4 (17)	5 (28) 40/60 1.2 (0.6–3.8) 0 (0) 1 (20)	19 (70) 26/74 2.9 (0.5–12.1) 2 (11) 3 (16)	0.005 0.61 0.62 1 1
Follow-up, months, median (IQR)	60.9 (24.7–92.2)	54.5 (22.3–94.5)	63.2 (29.5–90.9)	0.95
Death-censored graft failure, n (%)	8 (18)	0 (0)	8 (30)	0.01
Death, n (%)	16 (36)	8 (44)	8 (30)	0.31

DREMM, donor-specific antibody targeting a repeated antibody-verified eplet mismatch; ABMR, antibody-mediated rejection; TCMR, T-cell mediated rejection.

Regarding loci, all patients with DSA against a B and DQ antibody-verified eplet RMM (9 and 6 patients, respectively), experienced ABMR. In comparison, rates of ABMR with DREMM against A, Cw, and DR were 71%, 64% and 57%, respectively.

ABMR characteristics did not differ between the 2 groups, regarding the proportion of sub-clinical ABMR (29%) and the post-transplant time of ABMR diagnosis (median 2.4 months) (Table 4). Three patients with ABMR did not receive treatment for this episode of rejection. One patient was included in an Eculizumab trial and two patients were not categorized as ABMR according to the Banff classification used at that time. Noteworthily, there was no association between the risk of ABMR and any level of HLA incompatibility, antigenic, allelic or eplet, whether we considered repeated mismatches or not (Supplementary Table S2).

In univariate Cox model, factors associated with the survival without ABMR were the maximal MFI of the immunodominant DSA, the sum of the DSA MFI, and the presence of a DREMM (combined class I and II or class I but not class II alone) (Table 5). The number of mismatches and the presence of RMM, whatever the antigenic or eplet level, were not associated with an increased risk of ABMR. In the multivariate Cox model combining the MFI of DSA (either maximal value or sum of DSA MFI) and the presence of a DREMM, none of these factors was independently associated with the risk of ABMR. Given the association between DREMM and MFI of DSA (Table 3), we tested the presence of multicollinearity between these covariates, which was negative.

**TABLE 5:** Univariate and multivariate Cox model of variables associated with the risk of ABMR.

Immunological variables	N	HR	95% CI	*P*-value
	Univariate Cox model			
Immunodominant DSA maximal MFI, per 1,000 units increase	45	1.08	1.01, 1.14	0.015
Category of immunodominant DSA maximal MFI <3,000 3,000–10,000 >10,000	45	— 1.66 3.49	— 0.48, 5.75 1.25, 9.76	— 0.4 0.017
Immunodominant DSA Day-0 MFI, per 1,000 units increase	45	0.98	0.91, 1.07	0.7
Sum of DSA MFI, per 1,000 units increase	45	1.02	1.01, 1.03	0.007
Category of sum of DSA MFI <10,000 >10,000	45	— 3.35	— 1.42, 7.88	— 0.006
Number of antigenic mismatches, per unit	45	1.05	0.86, 1.28	0.6
Presence of repeated antigenic mismatches	41	0.85	0.31, 2.31	0.8
Number of eplet mismatches, per unit	45	1.03	0.98, 1.09	0.3
Presence of repeated eplet mismatches	44	0.72	0.21, 2.44	0.6
Presence of DREMM	45	3.37	1.25, 9.06	0.016
Presence of class I DREMM	45	2.54	1.10, 5.83	0.028
Presence of class II DREMM	45	2.00	0.87, 4.58	0.10
	Multivariate Cox Model			
1st model				
Immunodominant DSA maximal MFI, per 1,000 units increase	45	1.05	0.98, 1.13	0.13
Presence of DREMM	45	2.58	0.90, 7.34	0.076
2nd model				
Sum of DSA MFI, per 1,000 units increase	45	1.01	1.00, 1.03	0.087
Presence of DREMM	45	2.72	0.96, 7.67	0.059

HR, hazard ratio; CI, confidence interval; DSA, donor specific-antibody; DSA, donor-specific antibody; DREMM, donor-specific antibody targeting a repeated antibody-verified eplet mismatch; MFI, mean fluorescence intensity; Sum of MFI, sum of the MFI of each DSA the day of the maximal MFI of the immunodominant DSA.

**FIGURE 4:**
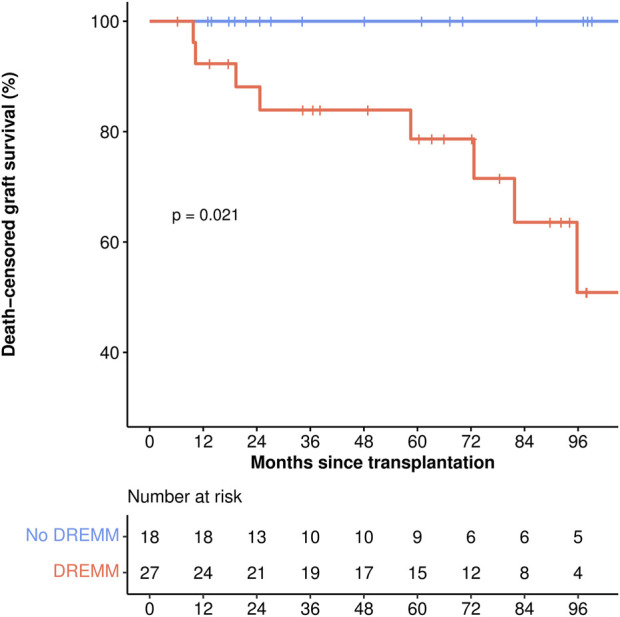
Death-censored graft survival. The red incidence curve corresponds to patients with DREMM, the blue curve to patients without DREMM. *p*-value corresponds to the log-rank test. DREMM, donor-specific antibody targeting a repeated antibody-verified eplet mismatch.

During the median follow-up of 61 months, 8 death-censored graft losses occurred, all in the DREMM group, attributed to rejection in all but one case. Therefore, the death-censored graft survival was lower in patients with DREMM (log-rank test: *P* = 0.02) (Table 4; Figure 4). No difference was observed in overall graft survival or in patient survival (Supplementary Figure S2).

## Discussion

We have shown in the present study that patients undergoing a kidney retransplantation with DSA targeting a repeated eplet mismatch (DREMM) experienced a higher frequency of ABMR and graft loss compared to patients with DSA targeting non-repeated eplets. Analyzing the target of antibodies at the eplet level reveals a higher frequency of repeated mismatches than at the antigenic level. These repeated mismatches frequently constitute the target of DSA against the second donor. Moreover, these DREMM display higher values of MFI, and a higher frequency of persistence after transplantation, both characteristics associated with a higher risk of ABMR and graft loss [3, 32].

To identify the eplet targets of DSA, we conducted a complete analysis of Luminex Single Antigen Bead raw data in all pre- and post-transplant sera with DSA in conjunction with the HLAMatchmaker analysis of both the mismatches and the specificity of alloantibodies in the recipients at the three levels, antigenic, allelic and eplet. This approach enabled us to obtain a comprehensive immunological phenotype for all patients to study the respective roles of its components in the occurrence of ABMR and graft loss. We also had access to the complete histological reports of all graft biopsies, which we reanalyzed according to the most recent Banff classification [30] to achieve a more accurate and homogeneous analysis.

Previous studies, conducted before the development of tools allowing to study donor/recipient mismatches at the structural level, have focused on the role of antigenic mismatches and on the antigenic target of alloantibodies.

Concerning kidney retransplantations, several studies have shown an association between antigenic RMM (class I and/or class II) and the risk of graft loss [14–16, 18], while others have not [17, 19]. However, none of these studies provided information regarding the presence of DSA. Interestingly, Lucisano et al. found an association between antigenic RMM, risk of ABMR and graft loss only in the presence of preformed DSA directed against this RMM [20]. Our study yielded similar results but with a higher sensitivity afforded by the eplet characterization of mismatches and DSA.

In the context of preformed DSA, eplet mismatches alone have no negative prognostic value in contrast to non-sensitized patients for whom they increase the risk of *de novo* DSA occurrence and therefore of graft loss [21–23, 25]. In our study, the risk of ABMR is mainly due to preformed DSA, which explains the lack of association between eplet mismatches alone, the risk of ABMR and graft loss. However, identifying patients with preformed DSA that targets a repeated eplet mismatch allowed us to classify them into two categories with different risks of ABMR and graft loss in univariate analysis.

To date, the DSA characteristics associated with ABMR include the maximum historical MFI values [3], their ability to bind complement [33], and their persistence at 3-month post-transplantation [32]. Interestingly, DREMM displayed significantly higher MFI values and were more often persistent at 3 months than DSA targeting non-repeated eplets. We could not show an independent effect of DREMM on the risk of ABMR. This may be due to the little size of our population, since even MFI of DSA was not associated with the risk of ABMR. Given the association between DSA MFI and DREMM, multicollinearity between covariates was tested and was negative. However, these results may suggest that immunization triggered by a first graft is likely to be stronger and more sustainable than that driven by other sources, such as pregnancy or transfusion. This echoes our previous observations that long-lived alloreactive memory B-cells were preferentially induced by kidney transplants rather than by pregnancies or transfusions [34].

As already mentioned above, the main limitation of our study mainly arises from the small number of patients included, due to the exclusion of patients whose data analysis did not allow us to identify the eplet target of DSA. In addition, a high-resolution HLA typing, necessary to determine eplet mismatches, was not available for all first donors due to the age of the first transplants. However, the French HaploSFHI algorithm, developed from data on 60,000 French patients and validated on four European cohorts, enabled to impute the missing typing with a high degree of accuracy, particularly given that our patients originated from the same region than patients included to develop the algorithm [31].

Our study focused on B-cell eplets, yet recent data also suggest a role for T-cell epitopes in alloimmunization. T-cell epitopes are derived from donor allogeneic HLA molecules, presented by HLA class II molecules on the recipient’s activated B-cells to recipient T-cells in secondary lymphoid organs. This step of indirect presentation is critical for the initiation of the humoral response and the synthesis of alloantibodies. The number of donor T-cell epitopes capable of being presented by recipient B-cells to T-cells has been quantified through the PIRCHE-II score (Predictable Indirectly ReCognizable HLA Epitopes) [35]. This PIRCHE-II score is correlated to the number of B-cell eplet mismatches [26, 36], and is also associated with the risk of *de novo* DSA and graft loss [26, 36–40]. Interestingly, recent studies have shown that for HLA-sensitized transplant recipients but devoid of preformed DSA, the presence of shared T-cell epitopes between the donor and the previous immunizers increased the risk of *de novo* DSA and possibly of graft loss [41, 42].

In conclusion, our work suggests that DREMM is a potential new marker of pathogenicity for DSA. Our results warrant further larger studies to conclude on the usefulness of DREMM as a new marker of ABMR risk stratification. Indeed, even if transplantations with preformed DSA are avoided in most of centers, all allocation systems do not fully prevent the presence of DSA. In France, for example, the threshold of MFI for DSA ranges from 1,000 to 3,000 to block allocation of kidney graft and does not concern Cw, DQA and DP loci. Moreover, some highly immunized candidates to retransplantation can only be transplanted in the presence of preformed DSA with specific immunosuppressive protocols, such as imlifidase, and in this context, improving prediction of the immunological risk with new markers like DREMM is an important clinical purpose.

## Data Availability

The raw data supporting the conclusions of this article will be made available by the authors, without undue reservation.

## Footnotes

1
www.divat.fr

2
www.agence-biomedecine.fr/

3 Available at http://www.epitopes.net/

4
https://www.epregistry.com.br/

## Abbreviations

ABMR, Antibody-Mediated Rejection; DREMM, DSA targeting a Repeated Eplet Mismatch; DSA, Donor-Specific Antibody; HLA, Human Leukocyte Antigen; MFI, Mean Fluorescence Intensity; RMM, Repeated MisMatch; SAB, Single Antigen Bead; TCMR, T-Cell Mediated Rejection.
