# Supplementary material for: Donor-Specific Antibodies Targeting a Repeated Eplet Mismatch and Outcome After Kidney Retransplantation
Source: Transpl Int. 2024 Nov 29;37:13639. doi: 10.3389/ti.2024.13639 (PMC11637850; doi:10.3389/ti.2024.13639)
Supplement: Supplementary file 1 [file DataSheet1.PDF]

Supplemental Material

Table of contents

Supplemental Figure 1. Flow chart.

Supplemental Figure 2. Overall graft survival and patient survival.

Supplemental Table 1. HLA mismatches at the antigenic, at the allelic and at the eplet level.

Supplemental Table 2. Mismatches, repeated mismatches and risk of ABMR.

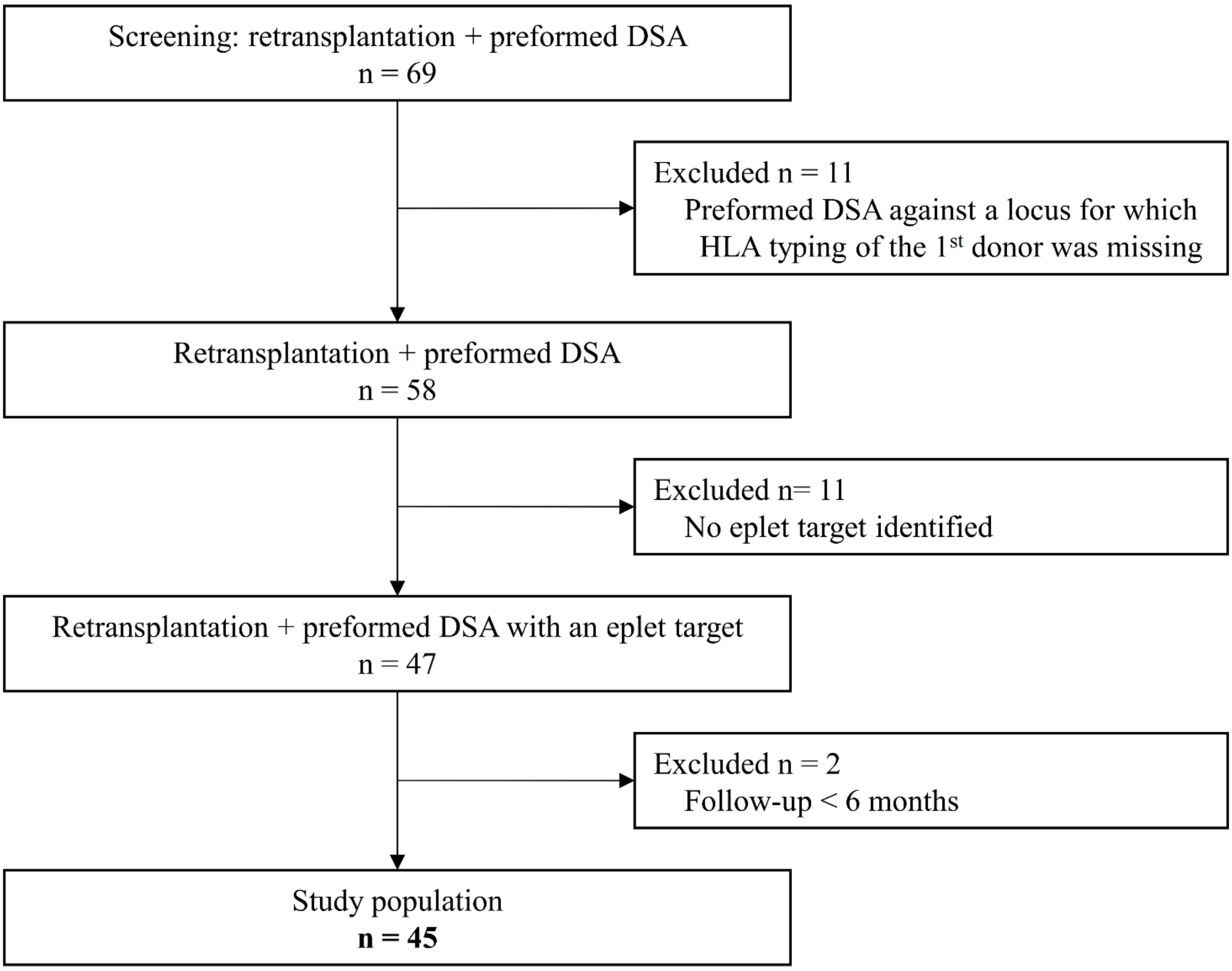

Supplemental Figure 1. Flow chart.
DSA, donor-specific antibody; HLA, human-leukocyte antigen.

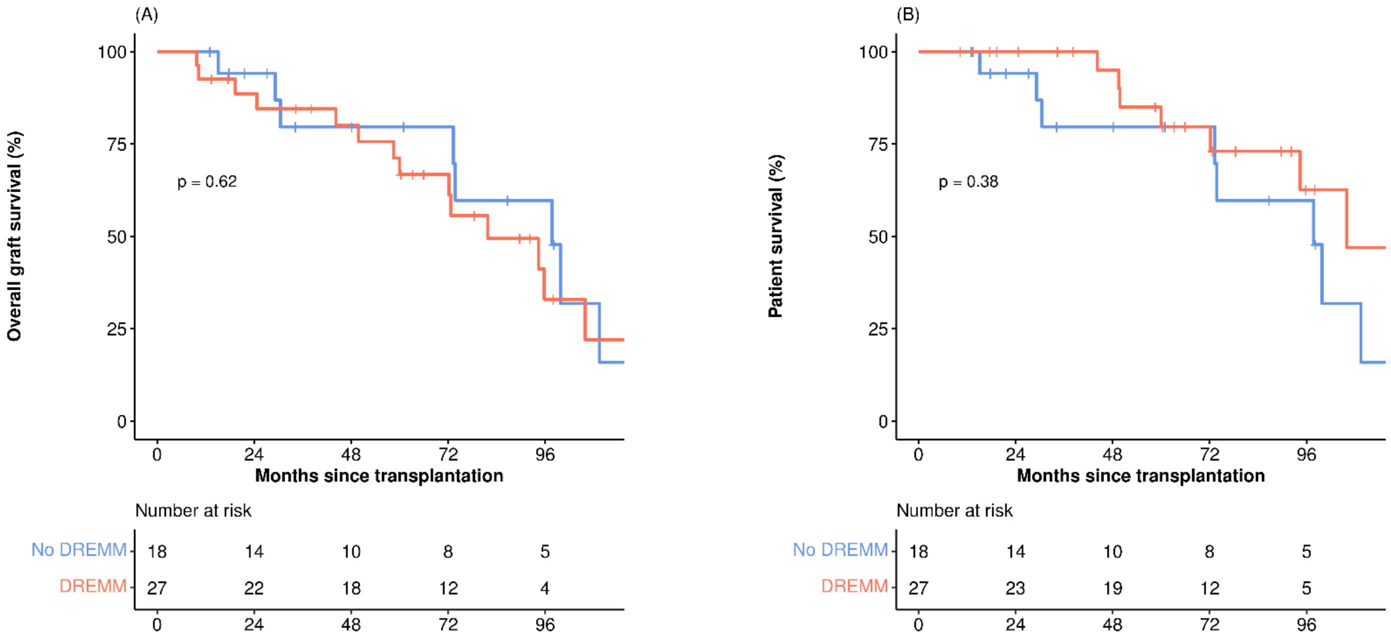

Supplemental Figure 2. Overall graft survival and patient survival.
(A) Overall graft survival. **(B)** Patient survival.
The red incidence curves correspond to patients with DREMM, the blue curves to patients without DREMM.
p-values correspond to the log-rank test.
DREMM, donor-specific antibody targeting a repeated antibody-verified eplet mismatch.

Supplemental Table 1. HLA mismatches at the antigenic, at the allelic and at the eplet level.

	**Total** n = 45	**No DREMM** n = 18	**DREMM** n = 27	**p**
**Antigenic MM**, mean ± SD	5.1 ± 2.3	5.1 ± 2.1	5.1 ± 2.4	0.93
Class I (A, B, Cw)	3.7 ± 1.4	3.7 ± 1.4	3.6 ± 1.5	0.86
Class II (DRB1, DRB3/4/5, DQB1)	1.4 ± 1.5	1.4 ± 1.2	1.4 ± 1.6	0.83
**Allelic MM**, mean ± SD	7.3 ± 3.2	7.2 ± 2.8	7.3 ± 3.5	0.82
Class I (A, B, Cw)	4.0 ± 1.5	4.0 ± 1.3	4.0 ± 1.6	0.79
Class II (DRB1, DRB3/4/5, DQA1, DQB1)	3.2 ± 2.3	3.2 ± 2.0	3.3 ± 2.6	0.93
**All eplet MM**, mean ± SD	42.9 ± 21.3	40.5 ± 18.8	44.4 ± 23.1	0.76
Class I (A, B, Cw)	27.0 ± 10.4	25.5 ± 7.6	28.0 ± 11.9	0.39
Class II (DRB1, DRB3/4/5, DQA1, DQB1)	15.8 ± 17.2	15.0 ± 15.5	16.4 ± 18.5	0.97
**AbV eplet MM**, mean ± SD	14.2 ± 7.2	13.1 ± 6.5	15.0 ± 7.6	0.44
Class I (A, B, Cw)	9.7 ± 4.2	8.7 ± 3.2	10.3 ± 4.7	0.18
Class II (DRB1, DRB3/4/5, DQA1, DQB1)	4.5 ± 5.2	4.3 ± 4.6	4.7 ± 5.6	0.89

DREMM, donor-specific antibody targeting a repeated antibody-verified eplet mismatch; MM, mismatch; AbV, antibody-verified.

Supplemental Table 2. Mismatches, repeated mismatches and risk of ABMR.

	**Total** n = 45	**No ABMR** n = 21	**ABMR** n = 24	***P* Value**
**Antigenic MM**	5.1 ± 2.3	4.9 ± 1.9	5.3 ± 2.6	0.77
Class I (A, B, C), mean ± SD	3.7 ± 1.4	3.6 ± 1.2	3.8 ± 1.6	0.82
Class II (DRB1, DRB3/4/5, DQB1), mean ± SD	1.4 ± 1.5	1.3 ± 1.3	1.5 ± 1.6	0.96
**Number of patients with RMM**, n (%)	10	5 (50)	5 (50)	
Patients without RMM, n (%)	31	14 (45)	17 (55)	1
**Allelic MM**	7.3 ± 3.2	7.0 ± 2.8	7.5 ± 3.6	0.76
Class I (A, B, C), mean ± SD	4.0 ± 1.5	3.9 ± 1.3	4.1 ± 1.7	0.75
Class II (DRB1, DRB3/4/5, DQA1, DQB1), mean ± SD	3.2 ± 2.3	3.1 ± 2.1	3.4 ± 2.5	0.94
**Number of patients with RMM**, n (%)	13	7 (54)	6 (46)	
Patients without RMM, n (%)	29	13 (45)	16 (55)	0.59
**Eplet MM (AbV)**	14.2 ± 7.2	12.8 ± 6.1	15.5 ± 7.9	0.34
Class I (A, B, C), mean ± SD	9.7 ± 4.2	8.8 ± 3.5	10.5 ± 4.7	0.28
Class II (DRB1, DRB3/4/5, DQA1, DQB1), mean ± SD	4.5 ± 5.2	4.0 ± 4.8	5.0 ± 5.5	0.92
**Number of patients with RMM**, n (%)	39	18 (46)	21 (54)	
Patients without RMM, n (%)	5	2 (40)	3 (60)	1

MM, mismatch; AbV, antibody-verified; RMM, repeated mismatch.
